# The roles of the nerve-immune axis in modulating bone regeneration

**DOI:** 10.1038/s41413-025-00476-3

**Published:** 2026-01-05

**Authors:** Yubin Zhao, Kaicheng Xu, Kaile Wu, Ziye Guo, Hengyuan Li, Nong Lin, Zhaoming Ye, Xin Huang, Jianbin Xu, Donghua Huang

**Affiliations:** 1 Department of Orthopedic Surgery, The Second Affiliated Hospital, Zhejiang University School of Medicine, Hangzhou, Zhejiang China; 2 Orthopedics Research Institute of Zhejiang University, Hangzhou, Zhejiang China; 3 Key Laboratory of Motor System Disease Research and Precision Therapy of Zhejiang Province, Hangzhou, Zhejiang China; 4 Clinical Research Center of Motor System Disease of Zhejiang Province, Hangzhou, Zhejiang China

**Keywords:** Bone, Neurophysiology

## Abstract

Bone is highly innervated, and its regeneration is significantly nerve-dependent. Extensive evidence suggests that the nervous system plays an active role in bone metabolism and development by modulating osteoblast and osteoclast activity. However, the majority of research to date has focused on the direct effects of peripheral nerves and their neurotransmitters on bone regeneration. Emerging studies have begun to reveal a more intricate role of nerves in regulating the immune microenvironment, which is crucial for bone regeneration. This review summarizes how nerves influence bone regeneration through modulation of the immune microenvironment. We first discuss the changes in peripheral nerves during the regenerative process. We then describe conduction and paracrine pathways through which nerves affect the osteogenic immune microenvironment, emphasizing nerves, neural factors, and their impacts. Our goal is to deepen the understanding of the nerve-immune axis in bone regeneration. A better grasp of how nerves influence the osteogenic immune microenvironment may lead to new strategies that integrate the nervous, immune, and skeletal systems to promote bone regeneration.

## Introduction

The interaction between the nervous system and bone tissue has garnered significant attention, establishing the nerve-bone axis as a crucial regulatory pathway in multifaceted contexts of bone development, homeostasis, disease, and regeneration.^1^ In embryonic skeletal development, innervation influences the development of long bones and flat bones through intramembranous ossification and endochondral ossification.^2–4^ The innervation first appears in the central part of the diaphysis and later extends to the metaphyses, which is related to vascularization.^5^ In addition, nerves play a key role in bone homeostasis by affecting the differentiation and development of mesenchymal stem cells, osteoblasts, osteoclasts, and chondrocytes.^6^ In disease contexts, such as osteoarthritis, sensory and sympathetic nerves infiltrate the typically avascular cartilage, contributing to inflammatory and pain processes through neuropeptides.^7–12^ In bone repair, the influence of nerves is particularly pronounced. Evidence suggests that intact innervation is crucial for effective fracture healing, with denervation resulting in callus formation that is mechanically weaker and less dense.^13^ The peripheral nervous system (PNS) regulates bone regeneration through neuropeptides and neurotransmitters released by injured or activated nerves, as well as nerve-resident cells.^14^

Bone regeneration is influenced not only by bone cells, stem cells, and other tissue cells, but also significantly by the immune microenvironment, as confirmed by numerous studies. The process of fracture healing occurs in four distinct phases: hematoma formation, soft callus formation, hard callus formation, and the remodeling of the hard callus.^15^ The initial phase is marked by hematoma formation triggered by the coagulation cascade and subsequent acute inflammation.^16^ Damage-associated molecular patterns from the extracellular matrix (ECM) and injured cells attract inflammatory cells to the injured site.^16^ Neutrophils are the first immune cells to arrive, followed by the recruitment of monocytes and macrophages through the release of inflammatory and chemotactic mediators, such as IL-6 and CCL2.^17^ The early inflammatory phase is essential for healing; disruptions or prolonged inflammation can hinder the process.^16^ Besides, the prolonged use of nonsteroidal anti-inflammatory drugs (NSAIDs) is associated with an increased risk of bone nonunion and delayed healing.^18^ Thus, inflammation is considered pivotal to successful fracture healing.^19^

Nerves are now known to play a crucial role in regulating the local immune microenvironment in various tissues such as muscles, skin, and the cornea. For instance, in injured skin and muscle tissues, the neuropeptide calcitonin gene-related peptide (CGRP), primarily released by sensory neurons, upregulates the gene Thbs1 in macrophages and neutrophils, which encodes thrombospondin-1 (TSP-1), through binding to the receptor activity-modifying protein 1 (RAMP1)-calcitonin receptor-like receptor.^20^ TSP-1, a multifunctional ECM protein, regulates tissue healing. Through this mechanism, CGRP inhibits neutrophil and monocyte/macrophage migration to injured tissue, accelerates neutrophil and inflammatory macrophage death in the presence of inflammatory cytokines, clears neutrophils, and promotes the polarization of macrophages into an anti-inflammatory, pro-repair phenotype.^20^ In diabetic corneas, Zhang et al.^21^ demonstrated that exogenous administration of vasoactive intestinal peptide (VIP) reduces the expression of pro-inflammatory cytokines IL-1β and CXCL2, while increasing the levels of anti-inflammatory cytokines IL-1Ra, IL-10, and CXCL5, ultimately facilitating wound healing in diabetic corneas.

Despite these insights, how nerves regulate immune responses during bone regeneration remains poorly understood. Recent findings indicate that nerve transection disrupts the local immune microenvironment during bone healing. For example, after tooth extraction, inferior alveolar nerve transection (IANT) alters the immune microenvironment, leading to increased neutrophil recruitment, changes in macrophage phenotype, and impaired bone remodeling.^22^ Furthermore, sympathetic nerve activation has been shown to create a local anti-inflammatory immune microenvironment following fracture.^23^ These observations suggest that the nerve-immune axis plays a significant role in bone regeneration as well.

In this review, we summarize current literature on the role of the nerve-immune axis in bone regeneration. We begin by discussing the changes in peripheral nerves during this process. We then highlight the critical role of the nerve-immune axis in bone regeneration and explain how the nervous system influences the osteogenic immune microenvironment. Critically, we delineate the roles of distinct nerves in modulating the osteogenic immune microenvironment and the effects of nerve-related factors on immune dynamics during different inflammatory phases. This review illuminates the multifaceted contributions of the nerve-immune axis to bone regeneration and lays the groundwork for further exploration of the complex interactions between the nerve-immune axis and bone regeneration.

## Changes in peripheral nerves in the bone repair process

**Fig. 1:**
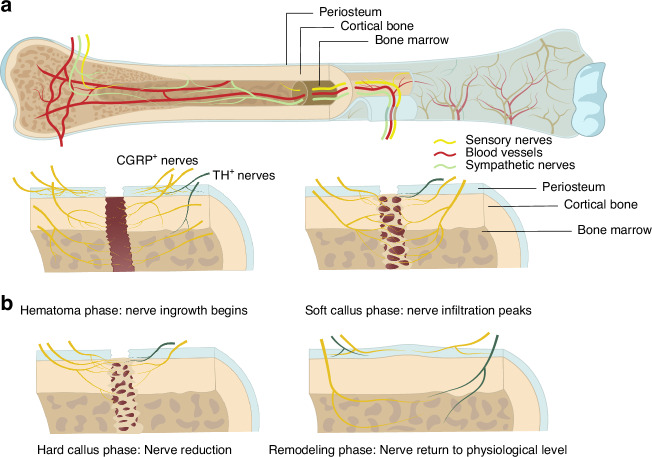
The distribution of nerves in normal bone tissue and after fracture. **a** Sensory nerves and sympathetic nerves enter the bone in parallel with the blood vessels, forming meshwork patterns in the periosteum, in which the sensory nerves are dominant. After penetrating the cortical bone, sympathetic nerves are more abundant. **b** Density of nerve fibers reaches the highest in the 3 days after fracture, and the nerves are mainly distributed in the periosteum. With the deposition of bone matrix, the nerve fibers gradually decrease and are limited to the outer fibrous capsule of the hard callus. Eventually, nerve fibers return to normal levels. Both CGRP^+^ and TH^+^ nerve fibers participate in the reinnervation after fracture, and the CGRP^+^ nerves contribute the most

Bone tissue is innervated by sensory, sympathetic, and parasympathetic nerves.^14^ Sensory neurons relay information from the periphery to the central nervous system (CNS), while efferent neurons transmit signals from the CNS to peripheral tissues.^24^ The periosteum exhibits the highest density of both sensory and sympathetic nerves, although sensory nerves are more abundant in this region. However, in mineralized bone and bone marrow, sympathetic nerves become more predominant^25^ (Fig. 1).

Sensory and sympathetic nerves enter the bone in parallel with the vasculature,^26^ forming distinct meshwork patterns in the periosteum^27^ (Fig. 1). These nerves descend through the outer connective tissue and inner cellular layers, which contain periosteum-derived mesenchymal stem cells, osteoclasts, and osteoblasts, all of which are crucial for bone maintenance and repair.^27–29^

Despite the high density of nerve fibers in the periosteum, the total number of sensory and sympathetic fibers is greatest in the bone marrow.^25^ Nerves penetrate cortical bone alongside blood vessels through nutrient canals, Haversian canals, and Volkmann’s canals, reaching the bone marrow and facilitating communication between the nervous and skeletal systems. This innervation plays a critical role in regulating bone metabolism and response to injury, influencing processes such as pain sensation, inflammation, and healing.^14^

When peripheral nerves at the fracture site are injured, retrograde injury signals are transmitted along the axons to the cell bodies, initiating nerve regeneration.^30,31^ In clinical practice, several classification systems have been developed for peripheral nerve injuries, among which the most widely accepted are the Seddon and Sunderland classifications.^32^ Seddon categorized nerve injuries into three types: neurapraxia, axonotmesis, and neurotmesis. Sunderland further expanded upon Seddon’s classification by dividing nerve injuries into first-degree to fifth-degree. First-degree injury corresponds to neurapraxia, while second-degree injury aligns with axonotmesis. Sunderland additionally introduced third-degree injury, in which not only is axonal disruption present, but partial damage to the endoneurium also occurs. Furthermore, Sunderland subdivided nerve injuries into fourth- and fifth-degree. Notably, in neurapraxia (first-degree injury), Wallerian degeneration does not occur because the underlying mechanism involves only a conduction block without true degeneration or regeneration, resulting in transient functional loss. In contrast, in second- to fifth-degree injuries, Wallerian degeneration takes place due to the disconnection of the distal segment from the neuronal cell body. Wallerian degeneration of distal axons leads to a compromised blood-nerve barrier, axonal and myelin degeneration, and subsequent clearance of myelin debris, initially by Schwann cells (SCs) and later by recruited macrophages.^30,31^ Concurrently, repair SCs proliferate and form temporary basal lamina tunnels that guide the regrowth of proximal axons toward their target tissues. Reinnervation precedes vascularization, ossification, and mineralization during bone healing.^33^

In the early stage of bone regeneration, prior to ossification, nerve fibers sprout in a dendritic manner from the periosteum, reaching their peak density in the first 3 days post-fracture before gradually declining. As bone matrix deposition progresses, nerve fibers become confined to the outer fibrous capsule of the hard callus. The predominant nerve fibers involved post-injury are CGRP^+^ peptidergic fibers, with a lesser contribution from TH^+^ sympathetic fibers.^33^ After bone repair, peripheral nerve levels typically return to baseline (Fig. 1). However, in cases of fracture nonunion, excessive innervation of the periosteum, cortical bone, and bone marrow can persist, often correlating with chronic pain.^34,35^

Peripheral nerves regulate bone regeneration through two primary mechanisms.^14^ First, neurotransmitters and neuropeptides derived from peripheral nerves directly act on bone-lineage cells, modulating bone regeneration by influencing the differentiation, proliferation, and viability of bone marrow-derived mesenchymal stem cells (BMSCs), osteoblasts, and osteoclasts. Additionally, nerve-resident cells, such as SCs and endoneurial mesenchymal cells, promote bone regeneration via paracrine factors or differentiation into bone-lineage cells.

## Nerve-immune axis during bone repair

**Fig. 2:**
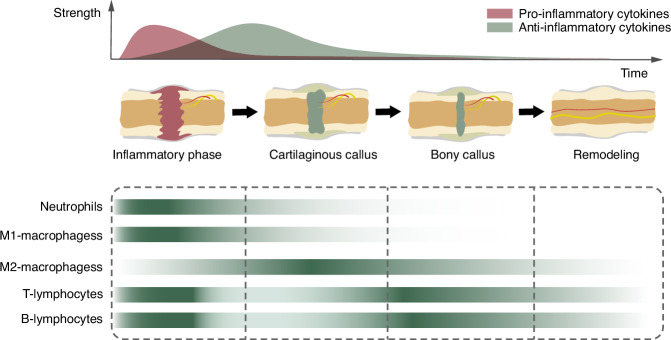
The diagram illustrates the dynamic fluctuations of pro-inflammatory and anti-inflammatory cytokines, as well as the changes in immune cells, throughout the progression of bone regeneration

The role of the immune microenvironment in the process of bone regeneration has been extensively studied. Inflammation is an immediate response to fracture, and the success of bone regeneration heavily relies on the efficiency of this initial inflammatory phase. The acute inflammatory response peaks within the first 24 – 48 h following injury and typically resolves within approximately 7 days.^18^ Both innate and adaptive immune responses are essential for bone regeneration.^15^ In recent years, the functions of neutrophils, macrophages, and T lymphocytes in bone regeneration have been well characterized.^16,19,36–38^ Furthermore, given the indispensable role of the immune microenvironment in bone regeneration, immunomodulation-based therapeutic strategies have been extensively investigated and systematically reviewed.^37–41^ For example, our previous studies not only demonstrated the critical role of M1 macrophage infiltration in the acute inflammatory response during the early stage of bone regeneration, but also developed a magnetically responsive hydrogel to facilitate macrophage polarization from the M1 to M2 phenotype, thereby ultimately promoting bone regeneration.^42^ During bone regeneration, the distribution of pro-inflammatory and anti-inflammatory cytokines, as well as distinct immune cell populations, is illustrated in Fig. 2. Immune cells are conventionally recognized to regulate bone-lineage cells, such as BMSCs, osteoblasts, and osteoclasts, through cytokine secretion. However, recent studies have highlighted the critical role of exosomes in intercellular communication. Exosomes derived from M2 macrophages promote the repolarization of M1 macrophages toward the M2 phenotype via the PI3K/AKT signaling pathway, and these repolarized macrophages further enhance the osteogenic differentiation of BMSCs.^43^ Exosomes derived from M2 macrophages promote osteogenic differentiation of BMSCs by carrying multiple microRNAs. Huang et al.^44^ demonstrated that M2 macrophage-derived exosomes enhance BMSC osteogenesis via miR-142-3p. Furthermore, these exosomes facilitate BMSC osteogenic differentiation and suppress adipogenic differentiation through the miR-690/IRS-1/TAZ axis.^45^ Additionally, miR-486-5p in M2 macrophage-derived exosomes stimulates osteogenic differentiation via the TGF-β/SMAD2 signaling pathway.^46^ In addition, Luo et al.^47^ demonstrated that miR-21a-5p in exosomes derived from M1 macrophages promotes osteogenic differentiation of MC3T3-E1 cells by targeting GATA2. Beyond non-coding RNAs, Pu et al.^48^ revealed that mechanical stimulation upregulates UCHL3 protein expression in macrophages, which interacts with SMAD1 in BMSCs to enhance osteogenic differentiation. Moreover, under regenerative conditions, overactivated macrophages can also secrete the bioactive lipid 11,12-EET through GSDMD pores.^49^ This lipid acts as a messenger to promote tissue regeneration.

**Fig. 3:**
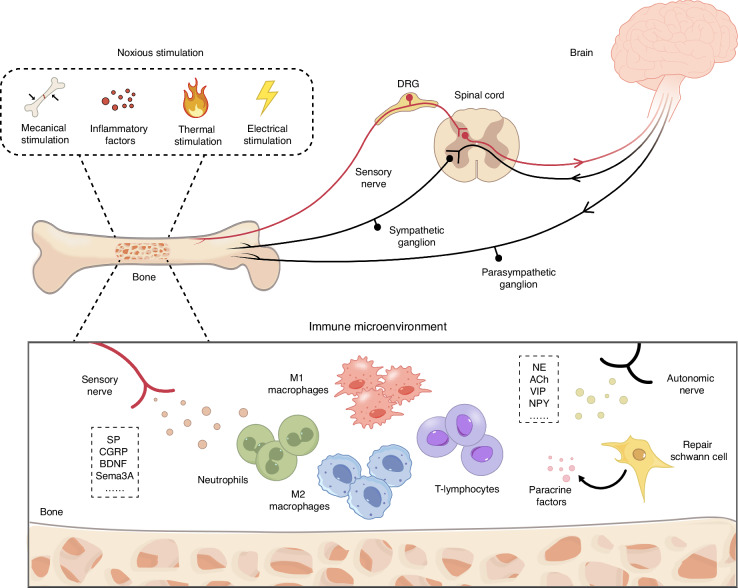
Illustration of two mechanisms by which the nervous system regulates the osteogenic immune microenvironment. (1) Conduction: peripheral sensory nerve endings, such as nociceptors, sense stimuli and transmit signals to the central nervous system (CNS) via ascending pathways. Different brain regions integrate, analyze, and regulate sensory information. Regulatory signals from the CNS are sent back to the periphery through descending pathways, modulating the activity of sympathetic and parasympathetic nerves. This activation or inhibition alters the osteogenic immune microenvironment through the release of neurotransmitters. (2) Paracrine pathway: after a fracture, damaged peripheral nerve axons are activated, leading to the direct release of neurotransmitters or neuropeptides. The release of nerve-related factors is not limited to nerve cells; other cells in the bone microenvironment, such as immune cells and stem cells (SCs), also secrete nerve-related factors and exosomes, affecting the osteogenic immune microenvironment through paracrine signaling

**Fig. 4:**
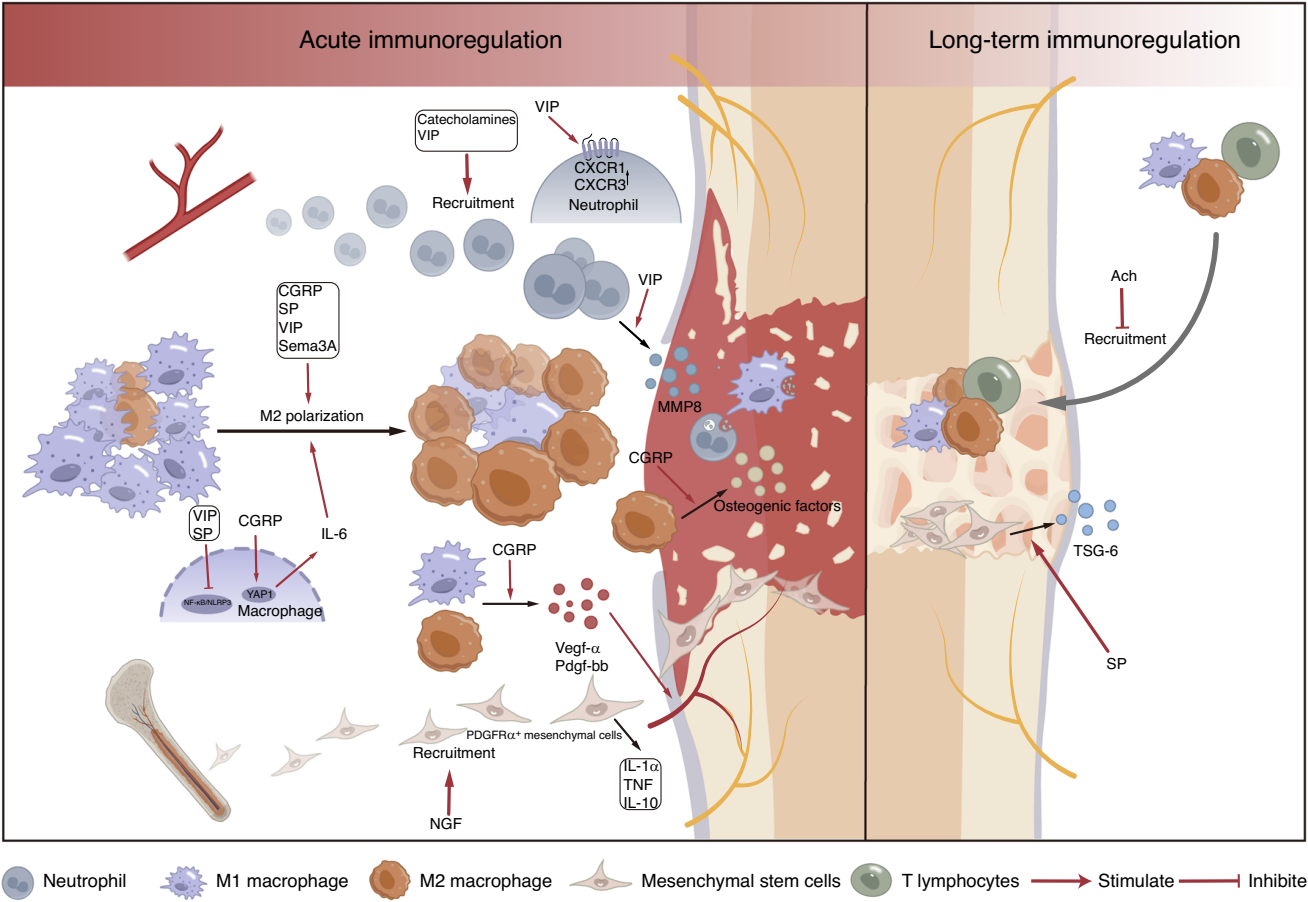
Illustration of the influence of nerve-related factors on the osteogenic immune microenvironment. During the acute inflammatory phase of bone regeneration, nerve-related factors influence bone regeneration by regulating the recruitment of immune cells and immunomodulatory stem cells, phenotypic switching of macrophages, and cytokine secretion. Mechanistically, VIP promotes the recruitment of neutrophils by enhancing the expression of neutrophil-related chemokine receptors. CGRP promotes IL-6 expression in macrophages by upregulating YAP1, thereby facilitating M2 macrophage polarization. Additionally, VIP and SP promote M2 macrophage polarization by inhibiting the NF-κB/NLRP3 signaling pathway. For long-term immunomodulation during bone regeneration, nerve-related factors may regulate bone regeneration through controlling immune cell recruitment and cytokine secretion by mesenchymal stem cells

The nervous system regulates the osteogenic immune microenvironment in two main ways: (1) Conduction pathway: mechanical deformation from fractures activates Aδ or C fibers, transmitting pain to the brain.^14^ CNS analyzes, integrates, and modulates ascending sensory information, then transmits descending signals via the autonomic nervous system (ANS), which comprises two branches, the sympathetic nervous system (SNS) and parasympathetic nervous system (PSNS), to peripheral target organs^50^ (Fig. 3). Skeletal is richly innervated by sensory nerves. Through viral tracing experiments and immunofluorescence, the pathways by which sensory nerves project from the periphery to the CNS have been well elucidated. Bone sensory neurons send a central projection via the L1–L6 dorsal root ganglia (DRG). Acute nociceptive stimuli from bone are transmitted to the CNS through the spinoparabrachial pathway, rather than the conventional spinothalamic tract or post-synaptic dorsal column pathways, which primarily relay cutaneous and visceral pain.^51^ In the conduction pathway, sensory nerves function to relay peripheral stimuli to the CNS. While the vagal ascending pathway is responsible for transmitting mechanical and chemical signals from visceral organs to the brain, whether skeletal afferent signals share this route remains unclear. The information transmitted by sensory nerves is processed by the CNS and then conveyed through sympathetic and parasympathetic nerves to modulate peripheral organs. Typically, ascending pathways first relay peripheral information to subcortical brain regions, such as the nucleus of the solitary tract (NTS), before transmitting sensory input to the parabrachial nucleus (PB), thalamus, hypothalamus, and hippocampus.^52–58^ These signals also project to higher cortical areas. For example, electrical stimulation of feline humeral nerves elicits evoked potentials in the primary and secondary somatosensory cortices, which are associated with Aδ fiber activation.^59^ The hypothalamus-ANS-bone efferent pathway has been extensively studied. For instance, leptin enhances sympathetic nerve activity by activating hypothalamic ventromedial hypothalamic (VMH) neurons.^60^ After injecting fluorescently labeled neurotropic pseudorabies virus into the rat femur, the virus labeled the lumbar and thoracic paravertebral chain ganglia at 4–5 days post-injection and labeled the intermediolateral column (IML), brainstem, midbrain, and specifically the locus coeruleus and hypothalamus, which are known to regulate SNS activity at 5–6 days post-injection.^61,62^ Additionally, following metaphyseal injections of the virus in the femur, viral-labeled cell bodies are localized to the sacral spinal cord’s intermediolateral column and central autonomic nuclei, which are limited only to parasympathetic preganglionic cell bodies, also confirming the existence of central-to-bone efferent pathways.^63^ Hu and Chen et al.^64,65^ comprehensively delineated a neural circuit connecting bone to the CNS and back to bone. Sensory nerves can sense bone density based on the concentration of PGE2, which is upregulated by factors regulating bone remodeling, such as PTH, mechanical loading, and TGF-β.^66–69^ When bone mass declines, such as in osteoporosis, more PGE2 is secreted by osteoblasts, which sends signals through EP4 in sensory nerves, inducing phosphorylation of CREB in the ventromedial nucleus of the hypothalamus (VMH). Activation of CREB signaling in the hypothalamus inhibits the tone of sympathetic nerves.^64,65^ The ANS affects the osteogenic immune microenvironment mainly by secreting neurotransmitters or neuropeptides. (2) Paracrine pathway: post-fracture, the peripheral nerves release neurotransmitters and neuropeptides via injured axons.^70,71^ Besides, sensory neurons can detect inflammatory mediators and growth factors released by immune cells during the inflammatory phase (Fig. 3). This detection enhances the sensitivity of the peripheral terminals of sensory neurons, promoting the release of neurotransmitters and neuropeptides.^70,72–74^ Additionally, axon reflexes may enhance neuropeptide release through antidromic action potentials. Pain stimuli activate local nociceptors, generating action potentials that transmit signals to the CNS. Simultaneously, antidromic action potentials travel toward the periphery, releasing neuropeptides.^14^ Actually, nerve-related factors can not only be released by neurons, but also by some immune cells and cells in bone tissues. For example, macrophages can secret acetylcholine (ACh) and Semaphorin 3A (Sema3A), and osteoblasts can also secret Sema3A. Moreover, SCs, nerve-resident cells, can switch to a reparative phenotype, secreting paracrine factors that regulate the osteogenic immune microenvironment^75,76^ (Fig. 3). Therefore, nerve-related factors from cells in the bone microenvironment, including immune cells, activated axons, and SCs, affect bone regeneration by regulating the immune microenvironment (Table 1) (Fig. 4). Furthermore, the increased neuropeptides are differentially distributed during bone regeneration (Fig. 2).

**Table 1:** Different immune cells are regulated by different types of nerve-related factors at different inflammatory stages of bone regeneration

Immune microenvironment			Nerve-related factors	Roles	Method	
Acute immunoregulation	Neutrophils and macrophages	CGRP		CGRP-deficient mice exhibited inhibition of M2 macrophage polarization and elevated levels of inflammation, while administration of CGRP lentivirus rescued these effects^116^		Mice with tooth extraction
				CGRP exclusion prolonged M1 macrophage infiltration and suppressed the transition toward M2 macrophages^117^		Rat with mandibular defect
				CGRP promoted IL-6 expression in M1 macrophages via YAP1, thereby facilitating M2 macrophage polarization; CGRP upregulated the angiogenic factors VEGF-α and PDGF-BB in M1 and M2 macrophages, respectively^117^		in vitro
				CGRP suppressed osteogenic factor expression in M2 macrophages during the initial 3 days but enhanced their expression on days 5 and 7 via YAP1^80^		in vitro
				Absence of α-CGRP appeared to promote M2 macrophage polarization^97^		Ovariectomized mice with femoral fracture
		SP		SP Promoted M2 macrophage polarization^125^		in vitro
		Catecholamine		Chronic psychosocial stress elevated Ly6G^+^ neutrophils locally within fracture hematomas, thereby impairing endochondral ossification, and this effect was ameliorated by propranolol administration^141^		PTSD mice with femoral fracture
				Neutrophil-derived catecholamines likely promote their own migration to fracture hematomas through a paracrine mechanism^143^		PTSD mice with femoral fracture
		VIP		Exogenous VIP administration promoted M2 macrophage polarization and neutrophil recruitment^137^		Mice with tooth extraction
		Sema3A		Sema3A induced apoptosis in macrophages^130^		in vitro
				Sema3A decreased the level of Pro-inflammatory cytokines^131^		Mice with periodontitis
		SCs-derived exosomes		Promote M2 macrophage polarization^149^		Rat with calvarial defects
	Mesenchymal stem cells	NGF		The absence of the p75 receptor in PDGFRα⁺ calvarial stromal cells significantly reduced mesenchymal subclusters expressing high levels of inflammatory cytokines and suppressed macrophage activation^152^		Mice with a calvarial defect
				NGF prevented the transformation of BMSCs into a pro-inflammatory phenotype and enhanced the secretion of immunosuppressive molecules^153^		Mice with diabetic periodontitis
	Others	NPY		Expression of NPY and Y1R increased^184^		Mice with a femoral defect
		BDNF		BDNF administration didn’t lead to excess inflammation compared to the control group^185^		Beagle dog with tooth extraction
Long-term inflammatory regulation	T lymphocytes	CGRP and SP		CGRP and SP didn’t influence the number of T lymphocytes^97^		Ovariectomized mice with femoral fracture
		NGF		NGF attenuated CD3^+^ T cell infiltration^155^		Mice with diabetic periodontitis
		Ach		Cholinesterase inhibitor administration reduced T cell populations^197^		Rat with tibial defect
	Mesenchymal stem cells	SP		SP promoted TSG-6 secretion from BMSCs, thereby establishing an anti-inflammatory immune microenvironment^186^		Mice with a calvarial defect
	Others	Ach		Donepezil reduced the infiltration of macrophages and T-cells^197^		Rat with tibial defects

*CGRP* calcitonin gene-related peptide, *SP* substance P, *PTSD* posttraumatic stress disorder, *VIP* vasoactive intestinal peptide, *Sema3A* Semaphorin 3A, *SCs* Schwann cells, *NGF* nerve growth factor, *PDGFRα* platelet-derived growth factor receptor α, *BMSCs* bone marrow-derived mesenchymal stem cells, *NPY* Neuropeptide Y, *BDNF* brain-derived neurotrophic factor, *ACh* acetylcholine

Neuropeptides influence the immune microenvironment in two key ways: (1) Vascular modulation: neuropeptides like CGRP and SP act on endothelial and smooth muscle cells, promoting vasodilation, plasma leakage, and immune cell adhesion to the endothelium. This facilitates immune cell migration from the bloodstream to the injury site.^77–79^ (2) Direct immune regulation: neuropeptides directly affect immune cell behavior, influencing processes such as proliferation, differentiation, and migration. For instance, CGRP enhances M2 macrophage polarization through the Yap pathway, contributing to tissue repair.^80^ This dual mechanism underscores the complex role of neuropeptides in immune modulation.

## Effects of different nerves on the osteogenic immune microenvironment

### Sensory nerves

Sensory nerves play a role in suppressing inflammation (Table 2) and promoting repair during bone regeneration. Capsaicin is commonly used to selectively induce chemical ablation of sensory nerves due to its preferential targeting of unmyelinated and small-diameter myelinated sensory neurons.^81^ However, achieving selective sensory nerve injury is challenging, as most peripheral nerves are mixed. The inferior alveolar nerve (IAN), a branch of the mandibular nerve, is initially a mixed nerve. Before entering the mandibular foramen, it gives off the nerve to the mylohyoid, which innervates the mylohyoid muscle and the anterior belly of the digastric. Within the mandibular canal, the IAN continues as a purely sensory nerve, innervating the mandibular teeth and supporting structures.^82^ Thus, IANT is an appropriate model for investigating the functional roles of sensory nerves. The IAN transection model is well-established. Following anesthesia, the surgical side of the animal is positioned distally relative to the operator. A 2.5 cm incision is made along the inferior border of the mandible from the tip of the angular process, followed by separation of the masseter muscle and blunt dissection to expose the IAN, which is then transected using microscissors. Subsequently, the distal stump retracts into the mandibular canal, and the proximal stump should be thoroughly excised to prevent regeneration into the canal.^83^ Transecting the IAN disrupts the immune environment, leading to increased neutrophil recruitment, elevated TNF-α levels, decreased IL-10, and altered macrophage morphology, creating a pro-inflammatory state that hinders bone repair.^22^ Moreover, elevated levels of IL-10 and TNF-α are still observed at 8 weeks post-injury. By implanting CGRP-loaded microbeads, the negative effects of IANT on the osteogenic immune microenvironment can be reversed. Furthermore, blocking macrophages with anti-F4/80 antibodies eliminates the influence of CGRP, demonstrating that CGRP, released from sensory nerves, suppresses inflammatory response primarily through macrophage activity and its potential therapeutic application in enhancing fracture healing. Therefore, Sensory nerves release CGRP to act on macrophages in the osteogenic immune microenvironment to create an anti-inflammatory and pro-repair environment for bone regeneration.^22^ The regulatory role of CGRP derived from sensory nerves on macrophages has been extensively studied across various tissues. CGRP exhibits context-dependent dual roles in different organs and pathological conditions, demonstrating both beneficial and detrimental effects. In skin and muscle tissue repair, Lu et al.^20^ demonstrated that CGRP enhances macrophage efferocytosis to clear neutrophils during the inflammatory phase, thereby preventing excessive inflammation (often linked to impaired wound healing), while simultaneously promoting macrophage polarization toward a pro-repair phenotype to facilitate subsequent tissue regeneration. Following corneal injury, CGRP administration significantly reduces the number of M1 macrophages and the level of TNF-α in the cornea.^84^ Following spinal cord injury, restoration of macrophage responsiveness to CGRP promotes M2 macrophage polarization, thereby attenuating glial scar formation.^85^ In acute respiratory distress syndrome (ARDS), CGRP alleviates ARDS-associated pathological damage, inflammation, and oxidative stress by modulating macrophage balance through inhibition of the HIF-1α pathway, reducing the proportion of M1 macrophages while increasing that of M2 macrophages.^86,87^ In both temporomandibular arthritis and corneal inflammation, CGRP promotes macrophage polarization toward the M2 phenotype, thereby facilitating the resolution of inflammation.^88,89^ In endometriosis, CGRP drives macrophages toward a pro-endometriotic phenotype, characterized by impaired exocytosis and enhanced support for endometrial cell growth in a RAMP1-dependent manner.^90^ In microbial infectious diseases, CGRP appears to hinder therapeutic outcomes due to its immunosuppressive effects and is paradoxically targeted as a therapeutic focus.^91–93^ For instance, during chronic Staphylococcus aureus infections, pain triggers persistent CGRP release from nociceptors. This CGRP binds to the RAMP1 on CX3CR1^+^ tissue-resident synovial lining macrophages, suppressing chemokine secretion and thereby establishing an immune-tolerant niche favorable for bacterial persistence.^94^ Interestingly, in our recent study, we observed that the M2-polarizing effect of sensory neuron-derived conditioned medium on macrophages was not significantly diminished by the application of CGRP-neutralizing antibodies, likely attributable to the low physiological concentration of CGRP under physiological conditions. Furthermore, we demonstrated that Sema3A derived from sensory nerves promotes macrophage M2 polarization by downregulating XIAP expression, thereby suppressing XIAP-mediated ubiquitination and subsequent degradation of PAX6 in macrophages.^95^

**Table 2:** Impact of different nerves on the osteogenic immune microenvironment

Study	Nerve	Method	Results
Xu et al.^22^	Sensory nerves	IANT	Increase of neutrophil recruitment, increase of TNF-α Decrease of IL-10 Elongation of macrophage morphology
Xu et al.^95^	Sensory nerves	M13-AV-NK phage	Promoting sensory nerve-derived secretion of Sema3A regulates macrophage polarization toward the M2 phenotype
Liu et al.^23^	Sympathetic nerves	TBI	Increased infiltration of M2 macrophage Increased proliferation of anti-inflammatory myeloid cells Increased levels of IL-10
Niedermair et al.^97^	Sympathetic nerves	Sympathectomized mice	Decrease of CD8-positive cytotoxic T cells Decrease of CD4-positive helper T cells
Ribeiro et al.^105^	Sympathetic nerves Parasympathetic nerves	CSN	Inhibition of the infiltration of inflammatory cells Decrease of TNF-α levels and IL-6 levels

*IANT* inferior alveolar nerve transection, *Sema3A* Semaphorin 3A, *TBI* traumatic brain injury, *CSN* carotid sinus nerve

### Autonomic nerves

Sympathetic nerves play a crucial role in bone remodeling and homeostasis.^14^ Activation of the SNS has been shown to modulate the immune system, such as increasing CD4^+^CD25^+^FoxP3^+^ regulatory T cells in the bone marrow.^96^ Liu et al.^23^ discovered that within 14 days following a traumatic brain injury (TBI) combined with fracture, TBI promotes M2 macrophage infiltration in the callus and stimulates the proliferation of anti-inflammatory myeloid cells in the bone marrow, including Ly6C^low^ monocytes and CD206^+^ macrophages. Additionally, TBI enhances IL-10 levels in the serum through the hypothalamic-sympathetic nerve pathway, thereby supporting fracture healing. Additionally, β2-ARs regulate M2 macrophage polarization, while β3-ARs influence hematopoietic progenitor cell proliferation. The effects of sympathetic nerves on the osteogenic immune microenvironment have also been explored under pathological conditions. In ovariectomized mice, Niedermair et al.^97^ found that, compared to WT mice, the number of CD8-positive cytotoxic T cells and CD4-positive helper T cells derived from the hematoma post-fracture was significantly reduced in sympathectomized mice, while the number of macrophages remained unchanged. The depletion of CD8-positive cytotoxic T cells typically benefits wound healing, while CD4-positive T cells contribute to bone remodeling by producing osteoprotegerin. Niedermair et al. hypothesized that the reduction in CD4-positive T cells during the early stages of fracture healing in sympathectomized mice might account for delayed callus maturation. Therefore, after fracture, sympathetic nerves also create an anti-inflammatory microenvironment (Table 2) that is beneficial for bone regeneration. Additionally, the activation of the carotid baroreflex suppresses cardiac sympathetic nerve activity while stimulating parasympathetic drive.^98^ In contrast, activation of peripheral chemoreflexes triggers both sympathetic and parasympathetic responses.^99^ The anti-inflammatory effects of the PSNS have been demonstrated in various diseases, including endotoxemia,^100,101^ sepsis,^100,102^ arthritis,^103^ and other inflammatory and autoimmune disorders. In systemic lipopolysaccharide (LPS)-induced endotoxic shock, efferent vagus nerve stimulation activates catecholaminergic splenic nerves, leading to increased norepinephrine (NE) release. NE acts on choline acetyltransferase-expressing (ChAT^+^) T cells via β2-adrenergic receptors, enhancing ACh secretion. Subsequently, ACh binds to α7 nicotinic acetylcholine receptors (α7nAChR) on macrophages, suppressing the production of pro-inflammatory cytokines, including TNF, IL-1α, and IL-8.^104^ However, research in this area remains scarce in bone repair. Therefore, independently investigating the role of the PSNS in the osteoimmunological microenvironment represents a meaningful study. In a mouse model of periodontitis, Ribeiro et al.^105^ simultaneously activated the baroreflex and peripheral chemoreflex via the carotid sinus nerve (CSN) to investigate its effects on periodontitis-associated inflammation and alveolar bone loss. CSN stimulation inhibited the infiltration of inflammatory cells, decreased TNF-α levels in the gingival tissue and IL-6 levels in plasma, and reduced alveolar bone loss.

## Effects of nerve-related factors on the osteogenic immune microenvironment

### Acute immunoregulation of bone regeneration

#### Neutrophils and macrophages

Acute inflammation is essential to normal bone regeneration. Depletion of macrophages inhibits the formation of callus.^106–108^ Polymorphonuclear neutrophils and M1-polarized macrophages play a critical role in clearing dead cells and debris while simultaneously releasing key pro-inflammatory cytokines.^109,110^ As the inflammatory phase transitions into the repair phase, macrophages undergo polarization to the M2 phenotype. The role of M2 macrophages is pivotal in resolving inflammation, promoting angiogenesis, and facilitating the recruitment and differentiation of cells critical for bone regeneration, such as BMSCs and osteoblasts.^111^ In fact, macrophages are present in all stages of bone regeneration. During the bone remodeling phase, macrophages continue to play critical roles in tissue remodeling and the resolution of inflammation.^38^ Dynamic regulation of macrophage phenotypes has been recognized as a promising therapeutic approach to enhance bone regeneration. Indeed, numerous studies have demonstrated that various neuro-related factors can facilitate macrophage phenotype switching during bone regeneration. Although macrophages are involved throughout the entire bone regeneration process, we specifically focus on promoting M2 macrophage polarization as a modulator of acute immune responses in bone regeneration, as this phenotypic transition typically occurs during the shift from the inflammatory phase to the chondrocallus formation phase.

During the acute inflammatory phase of bone regeneration, extensive studies have demonstrated that nerve-related factors can influence the recruitment of neutrophils and macrophages, macrophage polarization, and cytokine secretion. Among these factors, CGRP has been the most extensively investigated. CGRP is the major neuropeptide of sensory nerves, and various immune cells, including T lymphocytes, B lymphocytes, macrophages, mast cells, and dendritic cells, express CGRP receptors.^112–115^ In a mouse model with implants placed in the maxillary first molar extraction sockets, Yuan et al.^116^ found that the lack of endogenous CGRP inhibits M2 polarization of macrophages, resulting in increased inflammation in alveolar osseointegration and ultimately hindering bone integration. However, this effect can be reversed by restoring CGRP through lentiviral transfection. After CGRP transfection, the expression levels of M2 macrophage markers (CD206 and Arg1) were significantly elevated on day 7 of bone regeneration in comparison to the CGRP-deficient group. CGRP-mediated macrophage phenotypic alterations also regulate angiogenesis during early-stage bone regeneration. Similarly, in rat mandibular defect models, Kong et al.^117^ discovered that IANT or the injection of a CGRP receptor antagonist inhibits early osteogenesis, angiogenesis, and M2 polarization of macrophages and prolongs M1 macrophage infiltration. In vitro experiments showed that CGRP enhances the ability of both M1 and M2 macrophages to promote angiogenesis by increasing the expression of Vegf-α in M1 macrophages and PDGF-BB in M2 macrophages. This enhances the effects of M1 macrophages on tube formation and endothelial cell proliferation, as well as the effects of M2 macrophages on type H vessel generation. Furthermore, CGRP was shown to boost IL-6 secretion in M1 macrophages by promoting the expression of YAP1, a transcriptional coactivator that directly interacts with the IL-6 promoter.^117^ IL-6 from M1 macrophages is crucial for promoting the phenotypic switch of M1 macrophages to M2 macrophages. Using a CGRP lentiviral vector to stably transfect M2 macrophages, Zhang et al.^80^ found that CGRP suppressed the expression of osteogenic factors in M2 macrophages via the Hippo-YAP pathway during the initial 72 h. However, by days 5 and 7, CGRP significantly promotes the secretion of osteogenic factors such as BMP2, BMP6, WNT10b, and OSM in M2 macrophages, thereby improving the osteogenic immune microenvironment. CGRP-induced M2 macrophages exhibited effects on osteogenic marker expression in MC3T3 cells that were consistent with these findings. Niedermair et al.^97^ investigated the impact of CGRP deficiency on the osteoimmune microenvironment under pathological conditions (osteoporosis). However, under osteoporotic conditions, CGRP deficiency unexpectedly increased the proportion of M2 macrophages. Substance P (SP) is typically released in conjunction with CGRP from activated sensory nerves.^118^ NK1R has the highest affinity for SP and serves as its primary receptor.^119^ While research on SP’s regulation of the osteogenic immune microenvironment is still limited, SP has been shown to regulate inflammation and immune cell function to maintain tissue homeostasis.^120^ SP facilitates the transition from the inflammatory phase to the reparative phase by promoting acute inflammation and M2 macrophage polarization, effectively shortening the duration of the inflammatory phase. In TAC1 KO and NK1R KO mice, the absence of SP and/or NK1R resulted in a reduced acute inflammatory response on day 3 post-injury, followed by sustained low levels of pro-inflammatory cytokines by day 10, similar to what is observed in diabetic wounds.^121^ The recruitment of immune cells, such as neutrophils and T lymphocytes, to the wound site is a critical step in the acute inflammatory response. SP has been shown to enhance lymphocyte proliferation, cytokine secretion by lymphocytes and macrophages, and the recruitment of lymphocytes, monocytes, and neutrophils, thereby amplifying the acute inflammatory response and shortening its duration.^122^ Moreover, SP is a potent driver of M2 macrophage polarization. Studies have shown that SP directly promotes the expression of M2 markers, such as Arg1 and CD163, through the NK1R/PI3K/Akt/mTOR/S6K signaling pathway, even in the presence of IFN-γ, a potent inducer of M1 macrophages.^123^ In diabetic wound models, treatment with SP increased the proportion of M2 macrophages, reduced TNF-α levels, increased IL-10, suppressed inflammation, and ultimately promoted wound healing.^124^ Zhou et al.^125^ further verified SP’s ability to promote M2 macrophage polarization in vitro and explored its underlying mechanisms. Among the signaling molecules that regulate macrophage polarization, NF-κB and nucleotide-binding oligomerization domain-like receptor protein 3 (NLRP3) are particularly important.^126,127^ Activation of the NF-κB/NLRP3 signaling pathway promotes M1 polarization.^128^ In LPS-induced bone marrow-derived macrophages, SP was found to inhibit NF-κB phosphorylation and NLRP3 activation, suggesting that SP promotes M2 macrophage polarization by downregulating the NF-κB/NLRP3 pathway.^125^ Besides, Sema3A is a member of the semaphorin family.^129^ In vitro studies by Ji et al.^130^ demonstrated that Sema3A and its receptors are also expressed on monocyte-derived macrophages and play a key role in M2 macrophage polarization. During M-CSF-mediated macrophage polarization, the expression of Sema3A and its receptors, including NRP-1 and plexins A1, A2, and A3, is significantly upregulated, promoting the activation of the M2 phenotype. Additionally, Sema3A enhances the apoptosis of monocyte-derived macrophages without affecting their migration or phagocytic functions. In a periodontitis mouse model of orthodontic tooth movement, Satomi et al.^131^ found that local administration of Sema3A suppressed the production of pro-inflammatory cytokines such as IL-1β, IL-6, IL-17, TNF-α, and IFN-γ, while increasing the expression of the anti-inflammatory cytokine IL-10, ultimately preventing bone loss associated with orthodontic tooth movement.

Although several nerve-related factors have been reported to possess immunomodulatory properties, they may exert immediate effects at physiological concentrations. However, excessive administration-induced alterations in the immune microenvironment might not significantly influence the ultimate outcome of bone regeneration. For example, VIP is typically released from sympathetic nerves and functions through the GPCR receptors VPAC1 and VPAC2.^132^ VIP can also be secreted by immune cells.^133,134^ VIP exhibits potent immunomodulatory properties. In neural repair, VIP suppresses neurodegeneration by inhibiting the production of pro-inflammatory mediators.^135^ Moreover, in tendon repair, Wang et al.^136^ demonstrated that VIP suppresses the expression of macrophage-derived inflammatory factors, promotes their polarization toward the M2 phenotype, and enhances macrophage efferocytosis by downregulating the NF-κB signaling pathway. Furthermore, VIP-treated macrophages significantly accelerated the migration of tendon stem/progenitor cells. In a mouse tooth extraction model, Azevedo et al.^137^ found that exogenous VIP administration promotes M2 macrophage polarization, evidenced by decreased expression of pro-inflammatory cytokines (IL-1β, IL-6, TNF) and increased levels of anti-inflammatory and regulatory mediators (IL-10, TGF-β1), along with a reduced overall macrophage count. However, the increased expression of M2 macrophages peaked on day 7 and then gradually decreased, suggesting that VIP regulates early inflammation. Additionally, VIP administration was associated with increased expression of neutrophil-related chemokine receptors such as CXCR1 and CXCR3, higher activity of neutrophil marker MMP8, and elevated neutrophil counts. VIP is naturally expressed during alveolar bone repair,^138–140^ where it increases the expression of osteogenic factors, transcription factors for osteoblastic differentiation, and activity markers. However, further administration of VIP appears to have minimal impact on bone healing, and the decrease of endogenous VIP expression is observed. Therefore, under normal physiological conditions, the existing VIP signaling may already be optimal for bone repair, and additional signaling does not significantly enhance the process.

Moreover, neutrophil recruitment appears to be closely associated with psychological stress. In a posttraumatic stress disorder (PTSD) mouse model, Haffner-Luntzer et al.^141^ found that, compared to the control group, PTSD mice exhibited increased neutrophil recruitment in the bone marrow during the early phase of fracture healing, along with enhanced infiltration into the fracture hematoma. Both CD8^+^ and CD4^+^T cell populations were significantly reduced, while macrophage levels showed no significant difference. Additionally, impaired callus formation was observed in the late healing stage. Treatment with the β-AR blocker propranolol ameliorated these effects, suggesting that PTSD influences the osteogenic immune microenvironment and disrupts bone regeneration through β-AR signaling. In addition, Heidt et al.^142^ discovered that chronic psychological stress activates sympathetic nerves to release NE, which acts on β3-AR in bone marrow cells, inhibiting the secretion of CXCL12. This promotes the release of neutrophils and inflammatory monocytes into circulation. Building on this, Tschaffon-Müller et al.^143^ conducted further research and found that PTSD, when combined with fractures, causes local neutrophil-derived catecholamines in the bone marrow to activate both α- and β-ARs on chondrocytes. This activation hinders the differentiation of chondrocytes into osteoblasts, ultimately inhibiting fracture healing. Moreover, neutrophil-derived catecholamines promote their own recruitment to the fracture hematoma through a paracrine mechanism, potentially enhancing CXCL1 release from mast cells and macrophages within the hematoma. Collectively, these findings demonstrate that chronic psychological stress activates the SNS, which drives neutrophil infiltration from bone marrow into the fracture hematoma via NE-mediated mechanisms. This disrupts the inflammatory balance and ultimately impairs bone regeneration. Furthermore, neutrophil-derived catecholamines may facilitate their autologous migration to the hematoma through paracrine signaling. In myocardial infarction, metoprolol has also been demonstrated to reduce infarct size by suppressing neutrophil recruitment and attenuating the inflammatory response.^144^

In addition to being regulated by various factors, immune cells are also critically influenced by exosomes in intercellular communication. Exosomes can be secreted by all types of cells, including immune cells and nerve cells.^145^ As the key component of the paracrine pathway, exosomes are considered to be the facilitators of intracellular and intercellular communication.^146^ It has been reported that SC-derived exosomes possess immunomodulatory properties.^147,148^ However, the broader roles and mechanisms of these exosomes during bone regeneration remain largely unexplored. To address this, Hao et al.^149^ developed a gelatin methacryloyl hydrogel (GelMA) for loading SC-derived exosomes, ensuring their stability and sustained release. In a rat critical calvarial defect model, hydrogel-encapsulated SC-derived exosomes (Exo/Hydrogel) were found to promote innervation during bone regeneration, particularly by significantly increasing the presence of CGRP-positive nerve fibers. Early innervation in the bone defect area is critical for transitioning the bone regeneration process from the inflammatory phase to the fibrovascular phase. For instance, CGRP-positive nerve fibers promote M2 macrophage polarization, which is crucial for early-stage bone repair.^80^ Additionally, the implantation of Exo/Hydrogel resulted in a marked improvement in the inflammatory environment at the bone defect site.^149^ This was evidenced by a gradual increase in M2 macrophages, a rapid decrease in M1 macrophages, and a significant reduction in pro-inflammatory cytokines such as TNF-α and IL-1β during the inflammatory stage. This modulation created a favorable immune microenvironment, facilitating effective bone regeneration. Exosomes secreted by nerve cells also have immunomodulatory effects. Simeoli et al.^150^ demonstrated that when peripheral nerves are injured, the cell bodies of sensory neurons in the ipsilateral DRG release exosomes containing miR-21. Subsequently, the exosomes can be phagocytosed by infiltrating macrophages, leading to an increase of pro-inflammatory phenotype (M1) macrophages and a decrease of anti-inflammatory phenotype (M2) macrophages. However, during bone regeneration, whether exosomes secreted by nerve cells also contribute to the creation of an anti-inflammatory immune microenvironment remains to be studied.

#### Mesenchymal stem cells

In bone regenerative therapies, mesenchymal stem cells facilitate tissue repair not only through intrinsic osteogenic differentiation and paracrine cytokine secretion but also by exerting immunomodulatory effects that synergistically enhance bone regeneration.^111^ Nerve growth factor (NGF) has been demonstrated to regulate the immunomodulatory functions of stem cells. NGF expression sharply increased during the early phase following bone injury, peaking on day 3 and gradually declining thereafter.^151^ It was demonstrated that early-phase NGF primarily originates from macrophages and (platelet-derived growth factor receptor α) PDGFRα⁺ mesenchymal cells, while OCN⁺ osteoblasts serve as the dominant source of NGF in later stages of bone regeneration. However, NGF derived from PDGFRα⁺ mesenchymal cells did not significantly influence bone regeneration.^152^ In contrast, macrophage-derived NGF emerged as a critical mediator of bone regeneration by promoting sensory nerve ingrowth via TrkA signaling, which may indirectly influence the osteoimmune microenvironment. Besides, Xu et al.^153^ demonstrated that macrophage-derived NGF promotes the migration of platelet-derived growth factor receptor α (PDGFRα)⁺ calvarial stromal cells to injury sites via their p75 receptor. The absence of the p75 receptor in PDGFRα⁺ calvarial stromal cells significantly reduced mesenchymal subclusters expressing high levels of inflammatory cytokines (e.g., IL-1α, TNF, IL-10) and suppressed macrophage activation (both M1 and M2 phenotypes), as evidenced by diminished expression of TNF-α and the macrophage marker F4/80. Although macrophages also express the p75 receptor on their surface, NGF treatment only induced a modest upregulation of CD206 expression.^154^ Under diabetic pathological conditions, the NGF-TrkA signaling pathway exhibits anti-inflammatory functions. NGF has been shown to prevent the transformation of bone marrow mesenchymal stem cells into a pro-inflammatory phenotype and enhance the secretion of immunosuppressive molecules such as TGF-β and IL-10 under high-glucose conditions in vitro.^155^ This effect can be suppressed by TrkA inhibition.

NGF exerts its biological functions by binding to either the high-affinity receptor TrkA or the low-affinity receptor p75.^156,157^ Numerous in vitro models have demonstrated that NGF can enhance the activity of innate immune cells.^158–164^ During the acute inflammatory phase of wound healing, NGF-mediated activation of innate immune cells contributes to the initiation of inflammatory and regenerative signaling cascades.^165^ In vivo inflammatory models, exogenous administration of NGF has been shown to attenuate tissue inflammation.^166–169^ Mechanistically, studies have demonstrated that NGF binds to TrkA in TLR-activated monocytes and modulates downstream TLR signaling by reducing NF-κB nuclear translocation, thereby limiting the production of pro-inflammatory cytokines.^170,171^ Additionally, TrkA activation further enhances the PI3K/Akt pathway, a critical anti-inflammatory signaling axis downstream of TLRs.^172^ Thus, TrkA signaling may constitute part of an endogenous negative feedback mechanism engaged upon immune activation to restrain inflammation. Following Achilles tendon injury, inhibition of TrkA activity alters local inflammatory signaling, shifting macrophages from a “regenerative” phenotype to a “pro-inflammatory” phenotype. In osteoarthritis, inflammatory stimuli upregulate p75 receptor expression in bone cells, which suppresses inflammation-induced bone resorption and exacerbation of bone destruction by inhibiting the NF-κB signaling pathway.^173^ However, in rheumatoid arthritis, inflammatory stimuli upregulate the expression of proNGF and its low-affinity receptor p75 in synovial tissues, leading to increased production of IL-1β, TNF-α, and IL-6 in fibroblast-like synoviocytes and promoting chronic synovitis.^174^ Moreover, in allergic asthma, overexpression of NGF exacerbates airway hyperresponsiveness, while reduced p75 expression has been shown to be protective.^175^ Bandoła et al.^175^ demonstrated that p75 deficiency in plasmacytoid dendritic cells mitigates the development of allergic asthma. Collectively, the direct regulatory effects of NGF on inflammation appear to depend closely on receptor expression patterns and the stage of disease progression.

#### T lymphocytes

Emerging experimental evidence has demonstrated that T cells can be observed within fracture hematomas during the early phase of fracture healing.^176^ Clinical studies also demonstrated that the proportion of T cells increases in early human fracture hematomas and shifts toward an activated state.^177^ Furthermore, Dar et al. ^178^ demonstrated that γδ-T cells increased during the first 3 days of bone regeneration, corresponding to the early inflammatory phase and the rapid accumulation of γδ-T cells within the fracture callus induced systemic inflammation, leading to increased intestinal permeability. Specifically, the Th17 cell-inducing taxon segmented filamentous bacteria drives expansion of intestinal Th17 cells, while CCL20 mediates their homing to the callus. Moreover, blocking the egress of Th17 cells from the gut or inhibiting their homing to the callus significantly impairs the healing process, demonstrating the pivotal role of Th17 cells in bone regeneration. Although T cell involvement in the early inflammatory response has been well-documented, their precise roles and functions remain incompletely understood. Thus, further investigation is warranted to elucidate how peripheral nerves modulate T cell activity to influence the acute inflammatory phase of bone regeneration.

#### Others

The osteoimmune microenvironment is modulated not only by the aforementioned factors, but also by other nerve-related factors that have been implicated in immunoregulation. However, their precise roles remain to be elucidated. Neuropeptide Y (NPY) is widely distributed in both the CNS and PNS.^179^ Several studies have shown that bone is innervated by sympathetic nerves that express NPY, and non-neural cells within the bone microenvironment, such as osteocytes, bone marrow cells, and endothelial cells, can also secrete NPY.^180–183^ In a femoral defect model, Alves et al.^184^ observed an increase in the transcription and protein expression of NPY and Y1R in the defect area during the inflammatory phase, a response not seen in sham-operated animals. This indicates that the NPY system is a specific response to bone injury, potentially playing a role in regulating the associated inflammatory response. Immunohistochemical analysis revealed that during this phase, the polymorphonuclear cells recruited to the defect site were the primary cells expressing both NPY and Y1R, suggesting an autocrine effect. However, NPY-positive nerve fibers were not observed at any time point in the defect area, and were instead found distributed alongside blood vessels within the bone tissue and bone marrow. Additionally, increased NPY protein expression during the inflammatory phase was also detected in the DRG. These findings indicate that the NPY system is activated in the inflammatory phase of bone regeneration, but the effects of NPY on the immune microenvironment deserve further investigation.

Same as the NGF, brain-derived neurotrophic factor (BDNF) belongs to the neurotrophin family and exerts its biological effects by binding to either its specific high-affinity receptor TrkB or the low-affinity receptor p75.^156,157^ Kiyota et al.^185^ observed that BDNF-induced periodontal tissue regeneration occurs without significant infiltration of inflammatory cells. In contrast, the control group showed noticeable inflammatory cell infiltration, suggesting that BDNF may facilitate bone and periodontal tissue regeneration by suppressing excessive inflammation.

### Long-term immunoregulation of bone regeneration

In bone regeneration, inflammation typically subsides after 1 week. Therefore, in this review, we categorize the modulation of the immune environment beyond 1 week as long-term inflammatory regulation of bone regeneration.

#### T lymphocytes

Although the critical role of T cells in bone regeneration is being increasingly recognized, how nerve-related factors regulate T cells to influence bone regeneration and the underlying mechanisms remain poorly understood, highlighting an important area for future investigation. Furthermore, not all nerve-related factors have been found to affect T cells under physiological or pathological conditions. For example, in a mouse model of osteoporosis, the deletion of CGRP and SP did not significantly affect the number of CD8^+^ cytotoxic T cells or CD4^+^ T cells, at 5, 9, and 21 days after fracture.^97^ In the diabetic periodontitis model, NGF attenuated CD3^+^ T cell infiltration and suppressed hyperglycemia-induced excessive inflammatory activation.^155^

#### Mesenchymal stem cells

In a study using a calvarial defect mouse model, Zhang et al.^186^ demonstrated that intravenous injection of SP reduced both systemic and local inflammatory responses. However, previous research indicated that both endogenous and exogenous SP have short half-lives, with endogenous SP nearly disappearing within 3 days post-injury.^187,188^ Despite this, inflammation remained suppressed 2 weeks post-injury, suggesting that SP influences the osteogenic immune microenvironment by mobilizing exogenous BMSCs, which possess immunoregulatory properties. Moreover, SP administration significantly enhanced the survival of BMSCs. At 12 weeks post-operation, the GFP signal from GFP^+^ BMSCs was markedly higher in the treatment group compared to the control group. By assessing TSG-6, an anti-inflammatory protein secreted by BMSCs, researchers found an increase in TSG-6 expression both locally and systemically, indicating that SP can establish an anti-inflammatory immune microenvironment indirectly.^186^ In the OA model, Kim et al.^189,190^ developed a self-assembled peptide-SP (SAP-SP) hydrogel to increase the residence time of SP. Five months after injection, SAP-SP remained in the joint and infiltrated the synovium and cartilage surfaces. SAP-SP downregulated the expression of pro-inflammatory cytokines such as IL-1α and IFN-γ, while upregulating anti-inflammatory cytokines such as IL-4. This reduced inflammation, inhibited chondrocyte apoptosis, and promoted cartilage regeneration. Additionally, SAP-SP increased the proportion of M2 macrophages in circulation.

#### Others

Although in other tissue healing processes, the use of Ach promotes tissue healing through its anti-inflammatory properties. For example, in a rotator cuff injury model in mice, compared to the control group receiving empty fibrin gel, a higher number of M2 macrophages were observed in both the ACh and pyridostigmine (PYR) groups at 8 weeks post-surgery. The increased retention of M2 macrophages at the repair site likely promotes tissue repair, leading to improved biomechanical properties in these groups. At both 4 and 8 weeks post-surgery, the stiffness of the ACh and PYR groups was significantly greater than that of the control group.^191^ The regulatory mechanisms of ACh in inflammation have been extensively studied. ACh has been reported to significantly inhibit the production of TNF and other pro-inflammatory cytokines in LPS-stimulated macrophages.^192^ Additionally, activation of α7nAChR has been found to inhibit the apoptosis of M2 macrophages,^193^ a process identified as a key anti-inflammatory mediator.^194,195^ When ACh binds to α7nAChR on macrophages and other immune cells, it triggers intracellular mechanisms that include inhibition of NF-kB nuclear translocation and activation of the JAK2/STAT3 pathway. Furthermore, ACh can interact with α7nAChR expressed on mitochondria to inhibit the release of mitochondrial DNA, thereby suppressing inflammasome activation and the subsequent production of pro-inflammatory cytokines such as TNF and IL-1β.^196^ However, during bone regeneration, ACh does not appear to exert a beneficial effect on the process. Administration of the acetylcholinesterase inhibitor Donepezil impaired bone regeneration in rat tibial defects, accompanied by a significant reduction in macrophage and T cell infiltration at 2 weeks post-fracture.^197^

## Conclusion

The mysteries of bone biology are being unraveled at an unprecedented pace, significantly deepening our understanding of bone regeneration. In this review, we highlight not only the critical roles of the immune and nervous systems in bone regeneration but, importantly, emphasize the intricate involvement of the nerve-immune axis throughout the bone regeneration process. The immune microenvironment plays a pivotal role during both the acute inflammatory phase and the subsequent long-term repair process of bone regeneration. Based on the classification of acute and long-term immune regulation during bone regeneration, we, for the first time, systematically describe and summarize the distinct roles of different nerves and nerve-related factors in modulating the osteoimmune microenvironment.

While substantial progress has been made in understanding how the nervous system influences bone regeneration through immune modulation, this field remains in its early stages. The full range of neuropeptides and neurotransmitters’ effects on the osteogenic immune microenvironment has yet to be thoroughly investigated, and the mechanisms governing the nerve-immune axis during bone regeneration require further study. Current research predominantly focuses on the transition from M1 to M2 macrophages. Given the role of adaptive immunity in bone regeneration, further investigation is needed into how nerves and nerve-related factors regulate adaptive immune cells such as T and B lymphocytes during fracture healing.

Moreover, this review has primarily examined how the nerve-immune axis contributes to bone regeneration, but it is also important to recognize that nerves can be influenced by the immune system. Neurons can express receptors for cytokines released by immune cells, suggesting a bidirectional relationship. Thus, during bone regeneration, the impact of the local inflammatory microenvironment on the nervous system and the reciprocal crosstalk between nerves and immunity warrant deeper exploration.

In summary, this review aims to provide a comprehensive overview of the nerve-immune axis in bone regeneration, offering insights that may aid in the development of novel strategies for enhancing bone repair and regeneration.
